# Vertical transmission of avian leukosis virus subgroup J (ALV-J) from hens infected through artificial insemination with ALV-J infected semen

**DOI:** 10.1186/s12917-017-1122-4

**Published:** 2017-06-29

**Authors:** Yang Li, Shuai Cui, Weihua Li, Yixin Wang, Zhizhong Cui, Peng Zhao, Shuang Chang

**Affiliations:** 1 College of Veterinary Medicine, Shandong Agricultural University, Tai’an, 271018 China; 2 China Animal Health and Epidemiology Center, Qingdao, 266032 China

**Keywords:** Avian leukosis virus subgroup J, Semen, Vertical transmission, Specific pathogen free, Artificial insemination, Progeny chicks

## Abstract

**Background:**

Avian leukosis virus (ALV) is one of the main causes of tumour development within the poultry industry in China. The subgroup J avian leukosis viruses (ALV-J), which induce erythroblastosis and myelocytomatosis, have the greatest pathogenicity and transmission ability within this class of viruses. ALV can be transmitted both horizontally and vertically; however, the effects of ALV infection in chickens—especially roosters—during the propagation, on future generations is not clear. Knowing the role of the cock in the transmission of ALV from generation to generation might contribute to the eradication programs for ALV.

**Results:**

The results showed that two hens inseminated with ALV-J-positive semen developed temporary antibody responses to ALV-J at 4–5 weeks post insemination. The p27 antigen was detected in cloacal swabs of six hens, and in 3 of 26 egg albumens at 1–6 weeks after insemination. Moreover, no viremia was detected at 6 weeks after insemination even when virus isolation had been conducted six times at weekly intervals for each of the 12 females. However, ALV-J was isolated from 1 of their 34 progeny chicks at 1 week of age, and its *gp85* had 98.4%–99.2% sequence identity with the *gp85* of ALV-J isolated from semen samples of the six cocks.

**Conclusions:**

Our findings indicated that females that were late horizontally infected with ALV-J by artificial insemination might transmit the virus to progeny through eggs, which amounts to vertical transmission.

## Background

Avian leukosis virus (ALV) is one of the major causes of disease in poultry, and commonly produces tumours in those infected. This virus is recognized by host cell specificity and is susceptible to the virus neutralization reaction, which is mainly associated with the envelope protein, *gp85*. ALVs belong to the genus *Alpharetrovirus* of the family Retroviridae [1], and are divided into 10 subgroups, A to J, according to their host range, cross-neutralization, and viral interference [2]. The subgroup J avian leukosis viruses (ALV-J), which induce erythroblastosis and myelocytomatosis, have a greater pathogenicity and transmission ability than the other subgroups.

ALV-J was first isolated in 1988 from meat-type chickens in Great Britain and it mainly induced myelocytomatosis and nephromas [3]. During the last 10 years, ALV-J has been reported in many areas of the world [4–9]. Some previous studies have demonstrated that this tumorigenic virus can cause immune suppression [5], growth retardation, and tissue tumours in infected fowl [10, 11], which can cause substantial damage to the poultry industry [12, 13].

ALV can be transmitted horizontally and vertically [14–16]; however, the effects of ALV infection in chickens, especially roosters, during reproduction are not clear [17, 18]. In the cock reproductive organs, the virus budding phenomenon has been observed through electron microscopy in all structures except germ cells [17]. This indicates that the virus cannot proliferate in the germ cells. Therefore, the rooster may infect other chickens through contact or mating and will act only as a carrier of the virus [19–21]. It is not clear whether hens, after mating or other contact with infected semen, can produce viremia, especially for further vertical transmission to future generations [3].

In this study, each embryonated chicken egg was intravenously infected with ALV-J, and semen was collected from cocks with persistent viremia and used to inseminate specific pathogen free (SPF) hens. This research explores the possibility that hens infected with ALV-J through cock semen transmitted this virus to their offspring, and further clarifies the role of the cock on the infection and spread of ALV-J in chicken flocks.

## Results

### Isolation and identification of ALV-J in semen

Seminal fluid was collected from six cocks with persistent viremia and inoculated separately into DF-1 cells for ALV-J isolation. All supernatants inoculated with semen samples were positive for the p27 antigen using an ELISA, and IFA detection was positive using the ALV-J-specific monoclonal antibody JE9. At the same time, all negative semen from ALV-J negative control cocks was inoculated into DF-1 cells for p27 and IFA. The results indicated that all six cocks with persistent viremia could release ALV-J into their semen.

### Comparison of hens artificially inseminated with the ALV-J-positive or -negative semen for their viremia, antibody responses, and p27 shedding

The results showed that no viremia was detected in any of the hens at 1–6 weeks after insemination with ALV-J-positive semen. The ALV-J antibody response was negative at 1–4 weeks after insemination for all 12 hens. The ALV-J antibody response was positive at 5–6 weeks after insemination in 2 hens, while the other 10 hens were negative for ALV-J. The results of the p27 antigen detection experiments from the cloaca swabs showed a different percentage of p27 positive hens until the end of the experiment at 6 weeks. Viremia was not detected in hens during this experiment; however, 6 of the 12 hens were p27-positive when samples were taken from cloacal swabs at 4, 5, and 6 weeks. Therefore, the control group was inseminated with semen that was negative for ALV-J; all samples were negative for virus viremia, an ALV-J antibody response, and the p27 antigen at 1–6 weeks after insemination. These results indicated that some hens were infected horizontally through artificial insemination.

### Comparison of hens artificially inseminated with the ALV-J-positive or -negative semen for p27 antigens in their egg albumens

Eggs were collected from hens that were artificially inseminated with ALV-J-positive or -negative semen, and at one week post insemination, we attempted to detect p27 in the egg albumen. The results indicated that p27 was detected in the albumen of 3 of 26 eggs (11.54%) collected from hens inseminated with ALV-J positive semen, and the S/*P* values were 0.256, 0.322, and 0.565, which were significantly higher than the 0.2 baseline (S:P ratios that are greater than 0.2 were considered positive for ALV). In contrast, all 26 eggs of the control group were negative for p27, and their ELISA S/*P* values were less than 0.04. This indicated that some hens shed virus antigens into their egg albumen.

### Vertical transmission was confirmed in SPF hens artificially inseminated with ALV-J-positive semen

**Fig. 1:**
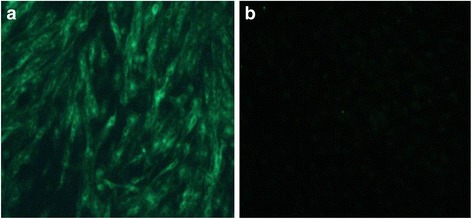
IFA detection for ALV-J of DF-1 cells of the progeny chicks. **a** Fluorescence from one of the progeny chicks’ plasma as detected by IFA using monoclonal antibodies JE9. **b** negative control

SPF hens were artificially inseminated, and after one week, eggs were collected for hatching. Hatched progeny chicks were tested for viremia and the presence of p27 by cloacal swabs. As indicated in Table 1, p27 was detected in cloacal swabs of approximately 20–30% of chicks from group 1 hens at 1 d, 1–3 weeks of age, whereas all samples were negative for p27 at 3 weeks of age for control group 2, indicating the possibility of vertical transmission for hens inseminated with ALV-J-positive semen. Importantly, ALV-J was isolated from 1 of 34 chicks at 1 week of age from hens inseminated with ALV-J-positive semen (Table 1). This was subsequently confirmed by IFA with the ALV-J-specific monoclonal antibody JE9 (Fig. 1) and *gp85* sequence analysis (see below). This is direct experimental evidence that hens artificially inseminated with ALV-J-positive semen may induce vertical transmission of the disease.

**Table 1:** The results of viremia and cloacal swab testing of progeny chicks after artificial insemination using the ALV-J positive and negative semen

	1d		1w		2w		3w	
	experimental	Control	experimental	Control	experimental	Control	experimental	Control
viremia	0/34	0/31	1/30^a^ (3.3%)	0/30	0/25	0/30	0/22	0/28
cloacal swabs	7/34 (20.6%)	0/31	9/30^b^ (30%)	0/30	5/25 (20%)	0/30	4/22 (18.2%)	0/28

^a^The chicken were dead at 10-days-old

^b^Three chickens were dead between 7 to 14 days of age in these nine chickens

### *gp85* sequence comparisons between ALV-J isolated from progeny, semen, and original challenge virus

The length of the *gp85* gene from ALV-J isolates is 921 bp. Sequence analysis demonstrated that *gp85* from ALV-J isolated from the progeny chick had a very high sequence identity of 98.4–99.2% when compared to viruses isolated from six semen plasma samples from cocks in group 1. Moreover, the sequence identity of ALV-J isolated from chicks was 98.1–99.0% when compared to the original ALV-J strain, 733, which was the original challenging virus for cocks. Furthermore, the ALV-J from six semen plasma samples had a *gp85* sequence identity of 97.6–98.8% when compared to the original strain (Table 2). This was strong evidence that the ALV-J isolated from the progeny chick came from the semen used to inseminate SPF hens.

**Table 2:** Sequence comparisons of gp85 of ALV-J isolated from the progeny, the semen, and original challenge virus

	733^a^	seminal fluid	progeny chicks
733^a^	-	97.6%–98.8%	98.1%–99.0%
seminal fluid	97.6%–98.8%	-	98.4%–99.2%
progeny chicks	98.1%–99.0%	98.4%–99.2%	-

^a^733 = original challenge virus

## Discussion

ALV can be transmitted horizontally and vertically, but congenital transmission through eggs is more important to leukosis outbreaks because most tumour cases occur in vertically transmitted or early horizontally infected chickens [22]. The critical roles of infected hens in transmission of ALV from generation to generation in chicken farms have been described and emphasized previously, especially for ALV-J eradication programs [3, 22], as Payne and Nair (2012) described that myelomatosis leukosis (ML) could occur in both congenital and early infected chickens. However, the role of cocks in ALV transmission is still not fully understood, and was described as “The role of males in the transmission of ALV is at best equivocal. Infection of the cock apparently does not influence the rate of congenital infection of progeny” and “The cock, therefore, is likely to act only as a virus carrier and source of contact of venereal infection to other birds**.**” Such conclusions were mainly based on early studies [15, 17, 19–21]. Even a more recent schematic description of ALV-J transmission failed to mention the role of infected cocks [3], as venereal infections are able to cause only late infections in females and unable to induce congenital infections in progeny.

The widespread outbreaks of ALV-J were reported in layers and in some Chinese indigenous breeds in recent years in China [23–25]. Studies of this epidemic indicated that the ALV-J infection of cocks might be responsible for the congenital infection of progeny. It was found that ALV was isolated from male chickens, but not female birds, in a large breeding farm, and their progeny developed tumours. In some indigenous chicken breed flocks, a high percentage of progeny were positive for ALV-J viremia when v + (i.e. viremia positive) rates were high in male breeders (e.g. >10%), but very low (e.g. <1%) in their female breeders (unpublished data). Such epidemic phenomena encouraged us to further elucidate the role of the cock in transmission of ALV from generation to generation, especially given that numerous breeding companies are interested in eradication programs for ALV.

In this study, six cocks maintained persistent viremia and shed the virus into their semen (they were termed ‘shedders’ and designated v + s+). The results indicated that the v + s + cocks might enable female breeders to transmit the virus to their progeny through their eggs. As Table 1 demonstrates, viremia was proven in 1 of 30 chicks hatched from eggs of 12 SPF hens artificially inseminated with v + semen, although all 12 hens remained viremia negative 6 weeks after insemination (Table 3). At the same time, p27 was detected in cloacal swabs from 20 to 30% of the chicks in the experimental group in the first 3 weeks, whereas none of the chicks from the control group was positive for p27. Sequence comparisons indicated that *gp85* of ALV-J isolated from the progeny chick and the semen had very high homology, with a sequence identity of 98.1–99.7% (Table 2), suggesting that ALV-J isolated from the one week old chick originated from the semen used for artificial insemination.

**Table 3:** The results of viremia, antibody response, and cloacal swab testing of hens after artificial insemination using the ALV-J positive and negative semen

week	group	viremia	ALV-J antibody response	cloacal swabs
1w	experimental	0/12	0/12	2/12
	Control	0/12	0/12	0/12
2w	experimental	0/12	0/12	5/12
	Control	0/12	0/12	0/12
3w	experimental	0/12	0/12	6/12
	Control	0/12	0/12	0/12
4w	experimental	0/12	0/12	6/12
	Control	0/12	0/12	0/12
5w	experimental	0/12	2/12^a^	6/12
	Control	0/12	0/12	0/12
6w	experimental	0/12	2/12^a^	6/12
	Control	0/12	0/12	0/12

^a^Hens are the same in two different times to produce antibody response

This is the first report to demonstrate that females infected late with ALV-J by artificial insemination may transmit the virus to their progeny through their eggs. It was reported that ALV does not multiply in germ cells of cocks [17], but ALV-J in the semen caused the venereal infection in hens, which further caused a congenital infection in progeny in this experiment. It was recognized that the genetics of different hosts and strains of ALV influence shedding and congenital transmission potentials after horizontal infection [18]. As such, further studies are needed to understand if the phenomena demonstrated in this manuscript would occur when different ALV-J strains and chicken breeds were involved. This study suggests that tests for male virus carriers would be as important as those for female breeders in eradication programs for ALV.

## Conclusions

Our findings indicated that females that were late horizontally infected with ALV-J by artificial insemination might transmit the virus to their progeny through their eggs, which amounts to vertical transmission of the virus.

## Methods

### Virus strains

ALV-J strain 733 was isolated from commercial layer hens and stored after replication in DF-1 cells (American Type Culture Collection, Manassas, VA) in our lab in China in 2012 [26]. The strain shows the typical myelocytomatosis, but no contamination of reticuloendotheliosis virus (REV), infectious bursal disease virus (IBDV), avian reovirus (ARV), chicken infectious anemia virus (CIAV), and Marek’s disease virus (MDV) by indirect immunofluorescence assay (IFA), reverse transcription polymerase chain reaction (RT-PCR), and dot-blot hybridization. There were 10^4^ TCID_50_ in each 0.1 mL of DF-1 cellular supernatant, which was diluted with PBS 1:10 before inoculating chick embryos.

### The infection experiment of ALV-J in cocks

SPF chicken embryos were obtained from the SPAFAS Co. (Jinan, China; a joint venture with Charles River Laboratory, Wilmington, MA, USA) and SPF chickens were normally hatched in our lab. Approximately 100 SPF chicken embryos were divided into 2 groups. All 11-day old chick embryos were intravenously inoculated with ALV-J of 1000 TCID_50_ (50 chicken embryos, group 1). The chicken embryos from the control group were inoculated with PBS (50 chicken embryos, group 2). For group 1, all hens were weeded out after eggs hatched and cocks were kept in a separate isolator. For group 2, all chickens were raised in another separate isolator. Blood samples were aseptically collected from all chickens in heparinized tubes at the age of 4, 8, 16, 20, and 24 weeks. For ALV viremia detection, DF-1 cells were inoculated with plasma samples from the chickens.

### Isolation and identification of the ALV-J in seminal fluid

Six cocks, which showed positive persistent viremia and negative viremia, were chosen for the collection of seminal fluid from group 1 after being raised to 24 weeks of age in isolators. The resulting seminal fluid was diluted 1:10 with Gibco Dulbecco Modified Eagle medium (DMEM, Life Technologies, Carlsbad, CA). The samples were then centrifuged at 10,000 *g* for 15 min at 4 °C. The supernatant was removed and filtered through a 0.22 μm filter (EMD Millipore, Billerica, MA), which was used to inoculate the DF-1 cells. The cells were cultured for 2 h at 37 °C, and the supernatant was replaced with fresh medium containing 1% foetal bovine serum [27]. The cells were incubated for an additional 7 d, and blind passages were performed for 2 generations over a total period of 21 d.

A 100 μL aliquot of cell culture supernatant was analysed for the presence of p27 from ALV using the Avian Leukosis Virus Antigen Test Kit (IDEXX Laboratories, Westbrook, ME). The ALV-positive supernatant samples were stored at −80 °C. The ALV-positive cells were fixed in an acetone-ethanol (3:2) bath for 5 min, and analysed using IFA with the JE9 anti-ALV-J monoclonal antibody [28] and an ALV-A/B antiserum [9], as previously described. Primary antibody reactivity was detected using a fluorescein isothiocyanate-labelled anti-mouse IgG antibody (Sigma-Aldrich, Saint Louis, MO). A drop of 50% glycerol was added to the coverslip, and the cells were observed using a fluorescence microscope.

### Detection of ALV-J infection in hens inseminated with the ALV-J infected semen

Semen samples were collected from six cocks with persistent viremia and mixed for artificial insemination of 12 SPF hens as the experimental group after the hens started producing eggs. Another 12 SPF hens were artificially inseminated with the semen collected from four cocks with no ALV-J infection as the control group. All hens of the two groups were bled once a week for 6 weeks after insemination. Plasma and serum were prepared for each individual hen for virus isolation in cell cultures or ALV-J antibody tests with ELISA antibody detection kits (IDEXX Laboratories, Westbrook, ME). At the same time, cloaca swabs were collected for each hen for p27 antigen detection with the Avian Leukosis Virus Antigen Test Kit (IDEXX Laboratories, Westbrook, ME). The eggs were collected to detect the presence of the p27 antigen from the egg albumen. Eggs were collected 1–3 weeks after insemination and hatched.

### Determination of ALV-J infection in progeny chicks

Anticoagulated blood samples from each chicken were collected at the age of 1 d. Subsequently, it took 1–3 weeks to inoculate DF-1 cells in order to conduct virus isolation tests as previously described. The cloacal swabs and meconium were analysed for the presence of the p27 antigen using the Avian Leukosis Virus Antigen Test Kit (IDEXX Laboratories), according to the manufacturer’s instructions. All samples collected were analysed in duplicate.

### Amplification and sequence analysis of viral RNA

The viral RNAs were extracted from the ALV strain 733, semen plasma samples, and ALV-J isolated from the progeny chicks using the Viral RNA Kit (Omega Bio-Tek, Doraville, CA). The purified RNAs were used for ALV detection by RT-PCR. The primers used for the amplification of the *gp85* cDNA from the ALV isolate were designed based on previous studies of representative ALV strains. The primers were as follows: F: 5′-GATGAGGCGAGCCCTCTCTTTG-3′; R: 5′-TGTTGGGAGGTAAAATGGCGT-3′. The PCR products were separated by electrophoresis on a 1% agarose gel. The *gp85* cDNA bands were purified from the gel using the EZNA Gel Extraction Kit (Omega), and ligated into the PMD-18 T plasmid (Takara Bio, Shiga, Japan). The vector was used to transform competent DH5α *Escherichia coli*. The sequence of the *gp85* cDNA was determined by a commercial service (Invitrogen, Shanghai, China). At least three independent RT-PCR experiments were performed for each sample to ensure the accuracy of the results. The sequence alignment was performed using with the Clustal application in the MegAlign program of DNAStar, version 7.01, software suite (DNAStar, Madison, WI, USA).

## Abbreviations

ALV: Avian leukosis virus
ALV-J: Avian leukosis virus subgroup J
ARV: Avian reovirus
CIAV: Chicken infectious anemia virus
DMEM: Dulbecco Modified Eagle medium
IBDV: Infectious bursal disease virus
IFA: Indirect immunofluorescence assay
MDV: Marek’s disease virus
ML: Myelomatosis leukosis
REV: Reticuloendotheliosis virus
RT-PCR: Reverse transcription polymerase chain reaction
SPF: Specific-pathogen-free
